# Protective effect of salidroside on streptozotocin-induced diabetic nephropathy by inhibiting oxidative stress and inflammation in rats via the Akt/GSK-3β signalling pathway

**DOI:** 10.1080/13880209.2022.2116055

**Published:** 2022-09-09

**Authors:** Delong Pei, Shengri Tian, Yanqiu Bao, Jun Zhang, Dongyuan Xu, Minhu Piao

**Affiliations:** a Department of Urology, Affiliated Hospital of Yanbian University, Yanji, Jilin Province, China; b Center of Morphological Experiment, Medical College of Yanbian University, Yanji, Jilin Province, China

**Keywords:** Anti-inflammatory, antidiabetic, *Rhodiola rosea*

## Abstract

**Context:**

Salidroside (SAL), one of the major glycosides isolated from the roots of *Rhodiola rosea* L. (Crassulaceae), has anti-inflammatory, antioxidant, and antidiabetic properties.

**Objective:**

Our study assessed whether SAL exerts a protective effect on streptozotocin (STZ)-induced diabetic nephropathy (DN) in rats via the Akt/GSK-3β signalling pathway.

**Materials and methods:**

Adult male Wistar rats were divided into three groups (*n* = 8): normal control, DN + vehicle, and DN + SAL. SAL (50 mg/kg/day, oral) was administered for 8 weeks. Biochemical and histopathologic examinations were performed to evaluate the therapeutic effects of SAL on oxidative stress, inflammation, renal function, and apoptosis.

**Results:**

SAL induced rats demonstrated ameliorated levels of FBG (20.53 ± 0.72 mmol/L vs. 26.02 ± 1.44 mmol/L), urine albumin excretion (27.00 ± 1.46 mmol/L vs. 41.00 ± 1.59 mmol/L), blood urea nitrogen (14.42 ± 0.70 mmol/L vs. 17.77 ± 0.72 mmol/L), and serum creatinine (112.80 ± 6.98 mmol/L vs. 159.00 ± 3.81 mmol/L) compared to normal control rats, along with the alleviation of renal pathologic changes by improving the irregular shape of glomeruli tissues. Biochemical analysis showed that SAL-treated animals displayed suppressed levels of serum inflammatory cytokines and kidney oxidative stress markers and attenuated apoptotic characteristics. Moreover, it increased the phosphorylation levels of Akt and GSK-3β in kidneys.

**Discussion and conclusion:**

The present study validated the involvement of the Akt/GSK-3β signalling pathway in renal improvement. These findings can form the basis to investigate the protective effect of SAL in DN in clinical trials.

## Introduction

The International Diabetes Federation (IDF) 2021 estimated that there are approximately 537 million adults worldwide living with diabetes and it is expected to increase to 783 million by 2045 (Facts and figures about diabetes 2021). Diabetic nephropathy (DN) is one of the most common long-term complications of diabetes with an average incidence of about 3% per year during the first 10-20 years after the onset of diabetes (Sulaiman 2019). Typical pathogenesis associated with DN includes glomerular hyperfiltration, hypertrophy, thickening of basement membrane, mesangial matrix accumulation, nodular glomerulosclerosis, and proteinuria (≥300 mg/day) leading to renal failure (DeFronzo et al. 2021). Progressive renal impairment can result in lipid disorders, hemodynamic abnormalities, release of inflammatory mediators, cytokines, oxidative stress, and apoptosis (Jing et al. 2017). The mechanism resulting in these alterations may include mitochondrial damage, overproduction of reactive oxygen species, or inflammation (Shati 2020).

Various signalling pathways are known to play a significant role in the initiation and development of DN (Shati 2020). The phosphoinositide 3 kinase (PI3K)/protein kinase B (Akt) signalling pathway may induce podocyte injury by altering its phenotype, which ultimately leads to the progression of DN. Glycogen synthase kinase 3 (GSK-3) is involved in the regulation of insulin cell signalling. Previously, studies have shown that high expression levels of GSK-3β is associated with decreased insulin sensitivity. At present, numerous studies are ongoing to establish the correlation between GSK-3β and type 2 diabetes (Jing et al. 2017).

Currently, the standard of care for DN includes glycemic optimisation, controlling blood pressure (BP) by exploiting the renin-angiotensin-aldosterone axis, and lipid-lowering agents (statins) (Guo et al. 2018). The Veterans Affairs Nephropathy in Diabetes (VA NEPHRON-D) study revealed that combination therapy with various BP-lowering agents does not prevent the progression of DN, rather it increases the chances of acute kidney injury, hyperkalemia, and hypotension. Hence, it is imperative to search for innovative therapies for DN, and a better understanding of the pathogenesis or signalling pathway is required, which could help us to identify new drug targets for DN (Chen et al. 2020).

Salidroside (SAL) is a phenylpropanoid glycoside obtained from the roots of *Rhodiola rosea* L. (Crassulaceae) (Zheng et al. 2015). The concentration of SAL extracted from the root extract is 2.7%. It has also been shown to protect cardiomyocytes from oxidative stress by activating the PI3K/Akt pathway (Zheng et al. 2015). Moreover, it also stimulates glucose uptake in skeletal muscle cells by activating AMP-activating protein kinase (AMPK) *in vivo* (Zheng et al. 2015). SAL exerts a variety of beneficial pharmacologic effects, including antioxidant (Xiao et al. 2014), anti-inflammatory (Lanza et al. 2001), and anti-apoptotic (Zhu et al. 2015) effects, and have shown to improve mitochondrial function (Zhang et al. 2015). Moreover, the role of SAL in preventing apoptosis has been reported in pheochromocytoma (PC12) cells, SH-SY5Y neuroblastoma cells, and cardiomyocytes. However, little is known about its effects on the apoptosis of proximal tubular epithelial cells induced by diabetes (Guo et al. 2018). Despite showing an array of pharmacologic properties, the suitability of SAL as a potential candidate for the treatment of DN remains unexplored till date. Therefore, the present study aimed to examine the effects and underlying mechanisms of SAL on streptozotocin (STZ)-induced DN using a rat model.

## Materials and methods

### Experimental animals

All experimental procedures were performed in accordance with International Guidelines for Care and Use of Laboratory Animals and were approved by the Animal Ethics Committee of Tongji Medical College, Huazhong University of Science and Technology. Twenty-four adult male Wistar rats (Yanbian University Laboratory Animal Centre) weighing 200–250 g were used in this study. Animals were housed and acclimatised in an air-conditioned animal facility under 12 h light/dark cycle with free accessibility to food and water. All experimental protocols and surgical procedures were performed in accordance with the recommendations of the Institutional Animal Care Committee of Yanbian University, Faculty of Medicine. Every effort was undertaken to minimise the pain and distress experienced by the animals. The catalog number and the manufacturer of all kits and reagents used in the current study are listed in Table 1.

**Table 1.** Key reagents table.

Name	Catalog #	Company
Streptozotocin	S0130	Sigma-Aldrich
Urinary albumin assay kit	E038-1-1	Jiancheng Biotechnology
Salidroside	–	the National Institute of Pharmaceutical and Biological Products
Triglyceride assay kit	A110-1-1	Jiancheng Biotechnology
Total cholesterol assay kit	A111-1-1	Jiancheng Biotechnology
Creatinine assay kit	C011-2-1	Jiancheng Biotechnology
Urea assay kit	C013-2-1	Jiancheng Biotechnology
IL-1β ELISA kit	SBJ-R0546	Nanjing SenBeiJia Biological Technology
TNF-α ELISA kit	SBJ-R0040	Nanjing SenBeiJia Biological Technology
Superoxide dismutase assay kit	A001-3-2	Jiancheng Biotechnology
Malondialdehyde assay kit	A003-1-2	Jiancheng Biotechnology
DeadEnd Fluorometric TUNEL System	G3250	Promega
Bcl-2	sc-7382	Santa Cruz Biotechnology
Bax	sc-7480	Santa Cruz Biotechnology
pSer473-Akt	sc-514032	Santa Cruz Biotechnology
Akt	sc-5298	Santa Cruz Biotechnology
pSer9-GSK-3β	sc-11757	Santa Cruz Biotechnology
GSK-3β	sc-7291	Santa Cruz Biotechnology
β-Actin	sc-8432	Santa Cruz Biotechnology

### Experimental design and sample collection

During the experimental period, all animals were supplied with a basal diet. DN was induced after 7 days by administrating a single-dose intraperitoneal (i.p.) injection of STZ (65 mg/kg) dissolved in freshly prepared citrate buffer (0.1 mol/L; pH 4.5) obtained from Sigma-Aldrich, (St. Louis, MO, USA). Animals with blood glucose level (BGL) ≥16.7 mmol/L and urine protein levels >30 mmol/L were used in the DN group. Rats were divided into three groups (*n* = 8): normal control (Con), DN + vehicle, and DN + SAL (50 mg/kg/day, oral) groups. Animals in the DN + SAL group were administered with SAL solution prepared in distilled water daily for 8 weeks (from week 5 to week 12). SAL (purity, >99%) was purchased from the National Institute of Pharmaceutical and Biological Products (Beijing, China). The rats in both the Con and DN + vehicle groups were injected with the same amount of citrate buffer (0.1 mol/L). Blood and urine samples were collected at 72 h after injection for the measurement of glucose and protein levels.

At the end of week 12, the 24 h urine samples were collected from all rats using special metabolic cages. Animals were killed after 20 min of the administration of 20% urethane (1 g/kg, i.p.), and their body weights were measured. Furthermore, rats were fixed on to an anatomic platform, abdominal fur was shaved after disinfection, and blood samples were drawn from the abdominal aorta by opening enterocoelia. The right kidney was excised, and the renal capsule along with upper and lower poles was removed. The remaining tissue was divided into three parts. The first part was rapidly frozen in liquid nitrogen and stored at −80 °C, the second part was immersed in 4% formaldehyde solution for generating paraffin slices, and the third part was fixed with 2.5% glutaraldehyde.

### Blood biochemical measurements

BGL was measured using a hand-held glucometer (Shanghai Qiangsheng Medical Equipment, Shanghai, China). Urinary albumin concentration was identified using the urinary albumin assay kit (Jiancheng Biotechnology, Nanjing, China). Triglycerides (TG) and total cholesterol (TC) were determined using a TG kit and a cholesterol kit (Jiancheng Biotechnology, Nanjing, China), respectively. The renal function was assessed by measuring serum creatinine (Scr) and blood urea nitrogen (BUN). Both the Scr colorimetric determination kit and the BUN kit were purchased from Jiancheng Biotechnology, Nanjing, China. All procedures were carried out in accordance with manufacturer’s instructions.

### Histologic analysis of renal tissues

After the animals were killed, a portion of the kidney tissue was rapidly fixed via immersion in 10% buffered formalin solution (pH 7.4) for 24 h. The fixed tissue was dehydrated in graded ethanol, embedded in paraffin, and cut into sections of 4 µm thickness. These tissue slices were incubated at 60 °C for 1 h; routinely dewaxed; rinsed; stained with haematoxylin and eosin (H&E), periodic acid-Schiff (PAS), and Masson; and then observed under an Eclipse 80i microscope (Nikon, Japan). Quantitative analyses of Masson’s staining for percentage of fibrotic area were carried out. Computer image analysis software (ImageJ) was used to quantitatively analyse the fibrotic area of Masson-stained tissue sections.

### Enzyme-linked immunosorbent assay

Enzyme-linked immunosorbent assay (ELISA) kits (Nanjing SenBeiJia Biological Technology, Nanjing, China) were used to determine the serum levels of interleukin-1 beta (IL-1β) and tumour necrosis factor-alpha (TNF-α). All assays were performed in accordance with manufacturer’s instructions.

### Biochemical analysis of oxidative stress markers and antioxidant enzyme activity

Normal saline was used to rinse and homogenise (100 mg tissue weight/mL) renal tissues. Furthermore, homogenates were centrifuged at 4000 rpm for 10 min at 4 °C. The levels of superoxide dismutase (SOD) and malondialdehyde (MDA) were assayed using the commercial colorimetric assay kits (Nanjing Jiancheng Bioengineering Institute, Nanjing, China). Superoxide anion radical is generated by xanthine/xanthine oxidase at pH 7.4. This radical reduces nitro blue tetrazolium into blue-formazan, which can be measured spectrophotometrically at 560 nm. One SOD activity unit can be defined as the amount of enzyme causing 50% inhibition in a 1 mL reaction solution per milligram of tissue protein. The SOD is expressed in terms of U/mg protein. Thiobarbituric acid (TBA) assay is the most commonly used method for determining the concentration of MDA in the homogenate. MDA forms a pink colour adduct with two molecules of TBA, which can be measured at 532–535 nm. Data were expressed in nmol/mg protein.

### Detection of apoptosis

The classic terminal transferase-mediated dUTP nick-end labelling (TUNEL) assay was performed for the detection and quantitation of apoptotic cells. The assay was performed in accordance with manufacturer’s instructions using commercially available kits (Promega, WI, USA). Paraffin sections were conventionally dewaxed, rehydrated with ethanol, and incubated with proteinase K (20 µg/mL) solution at room temperature for 8–10 min. About 100 µL of equilibration buffer was added followed by terminal deoxynucleotidyl transferase (TdT) reaction mixture for 1 h. Slices were stained with propidium iodide (1 µg/mL), and apoptotic tissues were analysed using confocal fluorescence microscopy.

### Western blotting analysis

Cell lysates of the frozen renal tissue samples were prepared by suspending at 4 °C for 30 min in phenylmethylsulfonyl fluoride (PMSF) followed by boiling at 97 °C for 10 min. Furthermore, the samples were centrifuged for 10 min (13,000 rpm at 4 °C), and the total protein concentrations in the supernatant were measured using commercially available bicinchoninic acid (BCA) protein assay kit (Thermo Fisher Scientific, Rockford, IL, USA). Western blotting analysis was performed on separated proteins immobilised on to a polyvinylidene fluoride (PVDF) membrane using antibodies against Bcl-2, Bax, pSer473-Akt (sc-514032), Akt, pSer9-GSK-3β (sc-11757), GSK-3β, and β-actin (Santa Cruz Biotechnology, Santa Cruz, CA, USA). Blots were incubated with horseradish peroxidase-conjugated secondary antibodies for 2 h. Immunoreactive bands were visualised using an enhanced chemiluminescence (ECL) kit (Pierce Biosciences, Rockford, IL, USA) and quantified by densitometric analysis performed using Quantity One software (Bio-Rad Laboratories, Hercules, CA, USA). In addition, primary antibodies were diluted 1:1000 in Tris-buffered saline with 0.1% Tween 20 detergent (TBST), and secondary antibodies were diluted 1:5000 in TBST.

### Statistical analysis

Data were calculated as mean ± standard error of mean (SEM). The significance was considered at *p* < 0.05. One-way ANOVA and two-way ANOVA tests with subsequent *post hoc* Tukey’s test were used for multiple comparisons. Significance was considered at *p* < 0.05.

## Results

### Effects of SAL on body weight and blood biochemistry

Fasting blood glucose (FBG), serum TC, and TG levels were higher in the DN + vehicle group compared with the Con group, whereas the body weight was markedly decreased in the SZT-induced DN group (Table 2). As evident from Table 2, the treatment with SAL ameliorated hyperglycaemia (FBG, mmol/L: vehicle, 26.02 ± 1.44; SAL, 20.53 ± 0.72) and dyslipidemia (TC, mmol/L: vehicle, 2.06 ± 0.07; SAL, 1.63 ± 0.16) in rats with DN. Moreover, SAL induced a significant increase in the body weight of rats with DN relative to those in the vehicle-treated group (Table 2). In addition, this experiment also investigated the dose-dependent effect of SAL. No statistically significant difference was observed in the changes in blood glucose and blood lipids when treated with 100 mg of SAL when compared with those in the group treated with 50 mg of SAL (Table 3). Furthermore, when 50 mg of SAL was administered to normal rats, no increase in blood sugar and dyslipidemia was observed (Table 4).

**Table 2.** Effect of SAL on different parameters in 3 groups.

Group (*N* = 8/group)	NC	DN + Vehicle	DN + SAL
BW (g)	268.30 ± 7.44	175.00 ± 4.30^****^	226.80 ± 6.28^####^
FBG (mmol/L)	5.45 ± 0.11	26.02 ± 1.44^****^	20.53 ± 0.72^####^
TC (mmol/L)	1.44 ± 0.003	2.06 ± 0.07^****^	1.63 ± 0.16^####^
TG (mmol/L)	2.09 ± 0.04	2.48 ± 0.36**	2.27 ± 0.16^#^
UV (mL/day)	26.93 ± 0.72	42.10 ± 4.30^****^	33.70 ± 0.89^####^
UP (mmol/L)	14.47 ± 0.55	41.00 ± 1.59^****^	27.00 ± 1.46^####^
BUN (mmol/L)	7.57 ± 0.12	17.77 ± 0.72^****^	14.42 ± 0.70^##^
Scr (mmol/L)	75.94 ± 2.98	159.00 ± 3.81^****^	112.80 ± 6.98^###^
IL-1β (ng/L)	12.65 ± 0.48	17.78 ± 0.35^****^	14.92 ± 0.46^###^
TNF-α (ng/L)	152.50 ± 9.87	280.81 ± 9.34^****^	209.32 ± 13.14^##^
SOD (U/mg)	36.67 ± 0.57	27.20 ± 0.39^****^	32.87 ± 0.46^####^
MDA (nmol/mg)	5.23 ± 0.21	13.65 ± 0.31^****^	11.72 ± 0.29^###^

**p < 0.01; ****p<0.0001 versus the NC group; ^#^*p* < 0.05, ^##^*p* < 0.01, ^###^*p* < 0.001, ^####^*p* < 0.0001 versus the DN group. All values are presented as mean ± SEM.

BW: body weight; BUN: blood urea nitrogen; DN: diabetic nephropathy; DN + SAL: diabetic nephropathy group treated with salidroside; FBG: fasting blood glucose; IL-1β: interleukin-1β; N: number; MDA: malondialdehyde; NC: normal control; SAL: salidroside; Scr: serum creatinine; SEM: standard error mean; SOD: superoxide dismutase; TC: total cholesterol; TG: triglycerides; TNF-α: tumour necrosis factor-α; UP: urine protein; UV: urinary volume.

**Table 3.** Effects of different doses of SAL on biochemical parameters.

Group (*N* = 8/group)	FBG (mmol/L)	TC (mmol/L)	Scr (mmol/L)
DN + SAL (50 mg)	20.53 ± 0.72	1.63 ± 0.16	112.80 ± 6.98
DN + SAL (100 mg)	19.01 ± 0.66	1.51 ± 0.13	108.58 ± 6.80

All values are presented as means ± SEM.

DN: diabetic nephropathy; DN + SAL: diabetic nephropathy group treated with salidroside; FBG: fasting blood glucose; TC: total cholesterol; Scr: serum creatinine; SAL: salidroside; SEM: standard error mean.

**Table 4.** Effects of SAL on biochemical indexes of healthy rats.

Groups (*N* = 8/group)	FBG (mmol/L)	TC (mmol/L)	Scr (mmol/L)
NC	5.45 ± 0.11	1.44 ± 0.003	75.94 ± 2.98
NC + SAL (50 mg)	5.34 ± 0.07	1.47 ± 0.05	77.12 ± 1.16

All values are presented as means ± SEM.

NC: normal control; NC + SAL: normal control group treated with salidroside; FBG: fasting blood glucose; TC: total cholesterol; Scr: serum creatinine; SAL: salidroside; SEM: standard error mean.

### Effects of SAL on renal function

Urine volume (mL/day) and urinary concentration of protein (mmol/L) were increased in vehicle-treated rats with DN (Table 2). Moreover, BUN (17.77 ± 0.72 mmol/L) and Scr (159.00 ± 3.81 mmol/L) were increased in the DN group, indicating obvious renal impairment induced by STZ (Table 2). Notably, the treatment with SAL reversed these changes indicated by a decrease in BUN (14.42 ± 0.70 mmol/L) and Scr (112.80 ± 6.98 mmol/L), suggesting alleviation of renal damage in rats with DN (*p* < 0.05). The values of all urinary parameters are summarised in Table 2. In addition, the change in creatinine level was not statistically different when treated with 100 mg SAL in comparison with the 50 mg SAL group (Table 3). On treating the normal control group with 50 mg SAL, no abnormal creatinine level was observed (Table 4).

### Effects of SAL on histopathologic changes

**Figure 1.**
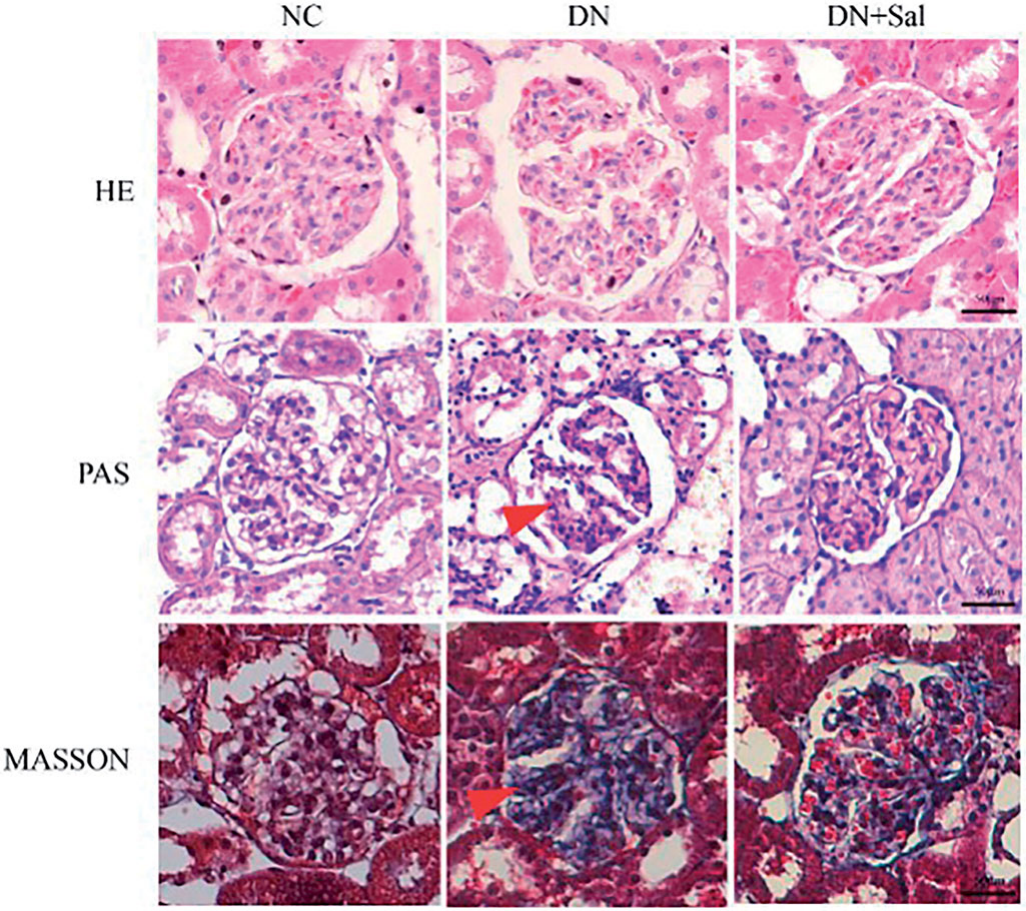
Effects of salidroside on the histopathologic changes of the kidney from rats with streptozotocin-induced DN (kidney tissue section, 400× magnification, scale bar = 50 μm). DN: diabetic nephropathy; DN + SAL: rats with DN treated with salidroside; HE: haematoxylin-eosin staining; NC: normal control; PAS: periodic acid-Schiff staining.

**Figure 2.**
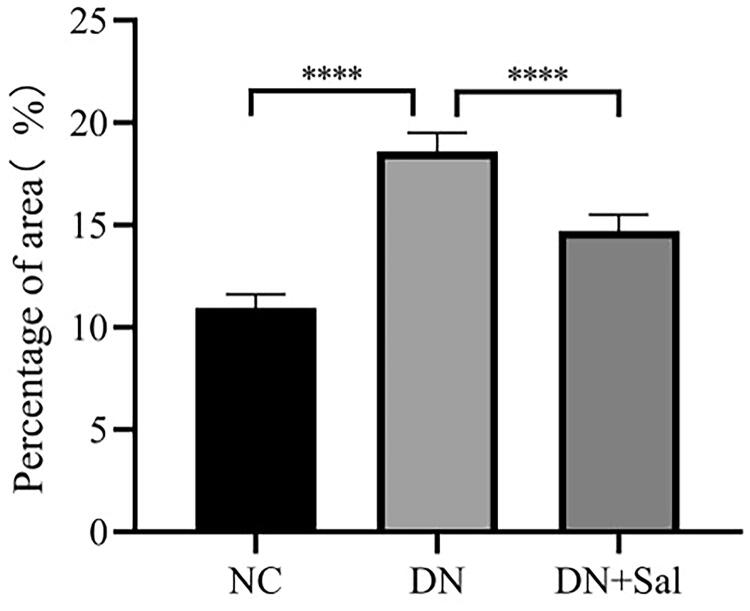
Quantitative analyses of Masson’s staining for percentage of fibrotic area ***p < 0.001. DN: diabetic nephropathy; DN + SAL: rats with DN treated with salidroside; NC: normal control.

Histologic changes in stained glomerular tissues were examined under a microscope. It can be clearly observed in H&E- and PAS-stained sections that the rats with DN showed abnormal glomerular architecture, swelling, and mesangial matrix expansion (Figure 1). In addition, Masson’s trichrome staining confirmed the increase in glomerular area, interstitial fibrosis, and mesangial expansion in the rats with DN (Figure 1). The treatment with SAL improved the irregular shape of glomeruli tissues, suggesting SAL ameliorates hyperplasia of kidney in the rats with DN. A graphical representation of the quantitative analyses of Masson’s staining for the percentage of fibrotic area is presented in Figure 2.

### Effects of SAL on inflammation and oxidative stress

The administration of SAL reversed the increase in serum levels of IL-1β and TNF-α. MDA levels were significantly decreased, but the activity of SOD was increased in renal tissues of the rats in the DN + SAL group compared with that of the rats in the DN + vehicle group (*p* < 0.05). The values of all inflammatory mediators and oxidative stress assays are summarised in Table 2.

### Effects of SAL on apoptosis

**Figure 3.**
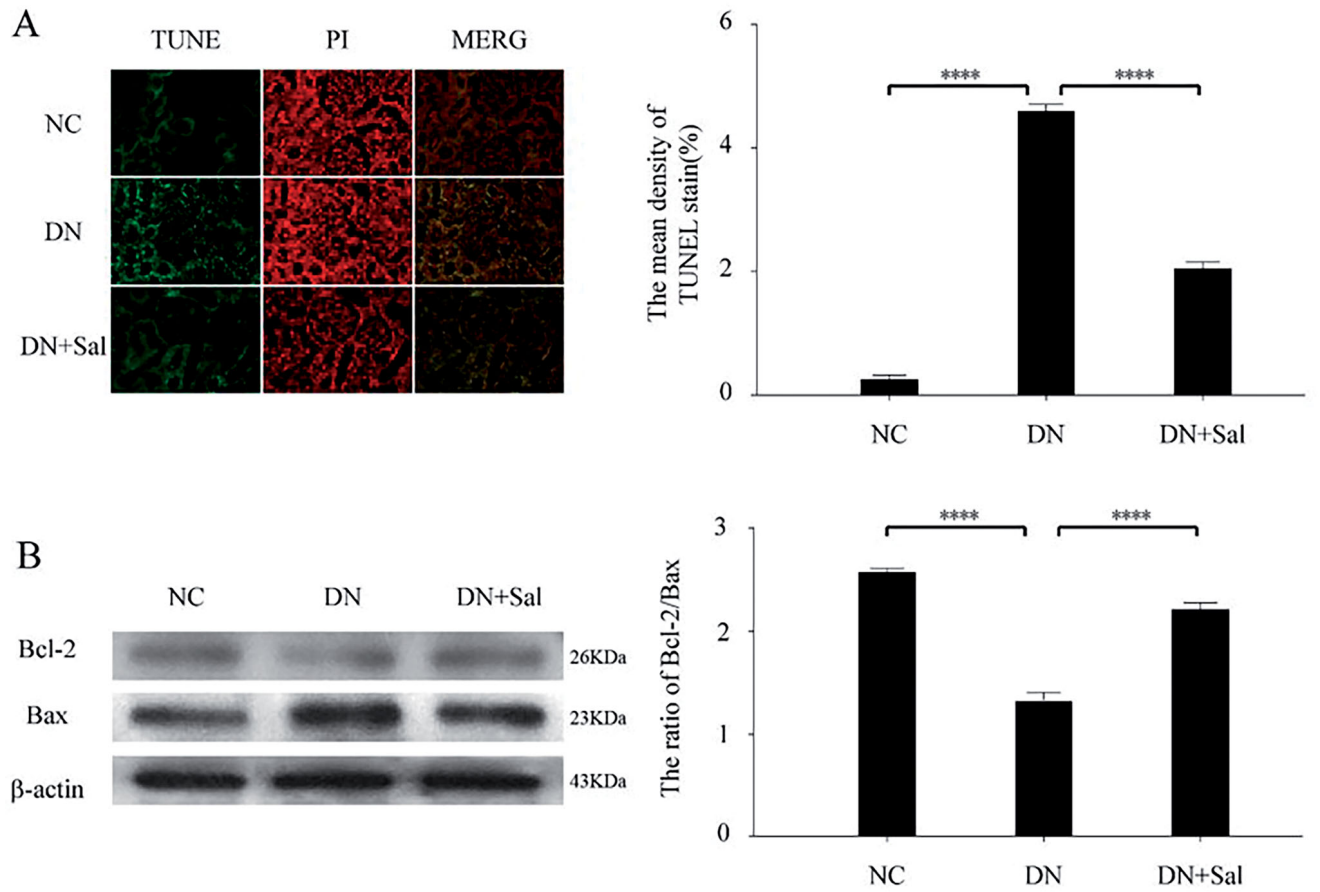
Effects of salidroside on apoptosis of the kidney from the rats with streptozotocin-induced DN. (**A**) TUNEL (green) and PI (red) staining was performed in the DN, DN + SAL, and NC groups. The TUNEL-positive stromal area quantification was shown by histogram. (B) Western blot analysis for the expression of Bcl-2 and Bax in kidney tissue and quantification of the Bcl-2/Bax levels. ****p < 0.0001. DN: diabetic nephropathy; DN + SAL: rats with DN treated with salidroside; NC: normal control; TUNEL: terminal dUTP nick-end labelling.

TUNEL staining analyses showed that SAL reduces the number of apoptotic cells in renal tissues. The cell death area was reduced with SAL treatment, signifying antiapoptotic effects (Figure 3(A). Furthermore, SAL significantly upregulated Bcl-2-to-Bax ratio in the rats with DN compared) with the vehicle-treated rats with DN (*p* < 0.05; Figure 3(B)).

### Effects of SAL on the Akt/GSK-3β signalling pathway

**Figure 4.**
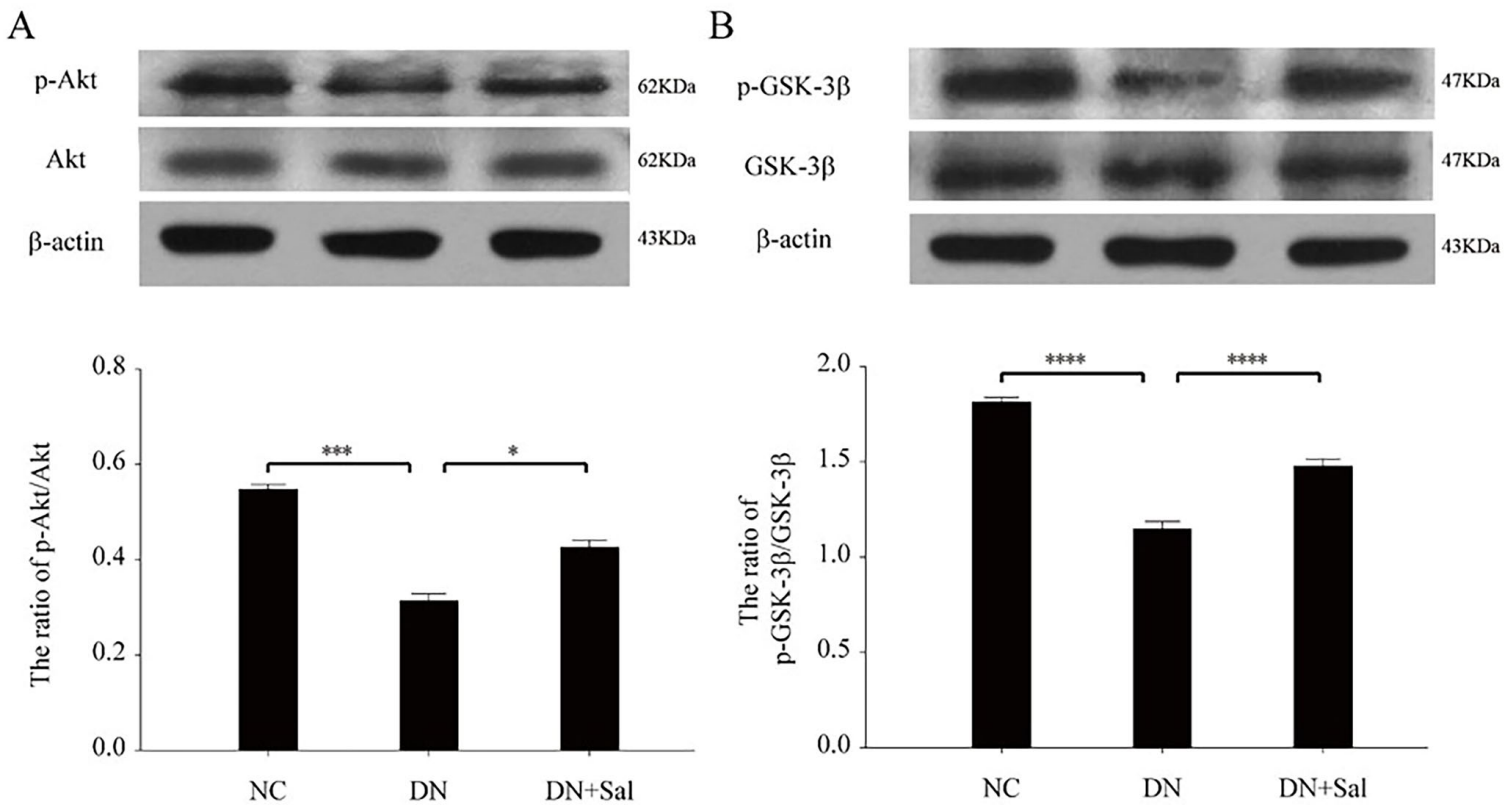
Effects of salidroside on the Akt and GSK-3β signalling pathway in the rats with streptozotocin-induced DN. (A) Western blot analysis for pSer473-Akt and total Akt in kidney tissue lysates from the DN, DN + SAL, and NC groups. The intensity of pAkt signal determined by scanning densitometry was normalised to total Akt. (B) Western blot analysis for pSer9-GSK-3β and total GSK-3β in kidney tissue lysates from the DN, DN + SAL, and NC groups. The intensity of p-GSK-3β signal determined by scanning densitometry was normalised to total GSK-3β. *p < 0.05, ***p < 0.001, ****p < 0.0001. DN: diabetic nephropathy; DN + SAL: rats with DN treated with salidroside; NC: normal control.

The treatment with SAL triggered phosphorylation of Akt (Figure 4(A)) and GSK-3β (Figure 4(B)), and the expression of pSer473-Akt and pSer9-GSK-3β was suppressed in the kidney of rats with DN but had no effect on protein levels of Akt and GSK-3β (*p* < 0.05). These results supported that SAL accentuates Akt/GSK-3β signalling via the activation of protein phosphorylation in the rats with DN.

## Discussion

The significant increase in understanding of DN pathogenesis and limitations of available treatments has led to requirement of novel antidiabetic drugs with higher efficacy and minimal side effects (Chen et al. 2020). The present study examined the therapeutic potential of a traditional Chinese medicine, SAL, in rats with STZ-induced DN. The findings from this study confirmed that the treatment with SAL ameliorated DN in rats with STZ-induced diabetes by inhibiting oxidative stress and inflammation via Akt/GSK-3β signalling pathway. The modulation of these processes by SAL was accompanied by changes in inflammatory (serum IL-1β and TNF-α), oxidative (SOD and MDA), and apoptotic (TUNEL assay and Bcl-2-to-Bax ratio) parameters.

The rats in the DN group showed significant hyperglycaemia, hyperlipidaemia, body weight loss, elevation of BUN, and degenerative changes in kidney cells, which may be attributed to dehydration and catabolism of fat and protein induced by the administration of STZ (Karuppusamy and Sasikala 2008). This indicates that the DN rat model was successfully constructed in the present study. Damage caused to insulin-secreting pancreatic β-cells results in absolute or relative deficiency of insulin, thereby decreasing protein synthesis in tissues (Graham et al. 2011). The SAL-treated group displayed a nephroprotective effect in rats because of stimulated glucose uptake in skeletal muscle cells by the activation of AMPK pathway along with enhanced cellular uptake of glucose (Li et al. 2008). SAL significantly reduced serum TG and TC levels in the rats with DN, possibly due to reduced activity of cholesterol biosynthetic enzymes or decreased lipolysis. This is in line with a recent finding which suggested that SAL inhibits *de novo* lipogenesis and cholesterol biosynthesis in atherogenic mice along with lowering of hepatic levels of glucose and accelerated fatty acid degradation (Song et al. 2021).

In the current study, structural changes were observed in the rats with DN in the form of increased glomerular index, abnormal glomerular architecture, interstitial fibrosis, swelling, and mesangial matrix expansion. However, the shape of glomeruli tissues was improved after the treatment with SAL, indicating the repairing effect of SAL on the structure of kidneys. DN in rats was accompanied by inflammatory processes; the administration of SAL led to a decrease in the levels of inflammatory factors (IL-1β and TNF-α) in the rats with DN, indicating protective effect of SAL. Also, SAL has been shown to exert anti-inflammatory effect and prevent brain edoema by reducing the expression of TNF-α and neutrophil infiltration in rats with cerebral ischemia-reperfusion injury (Han 2013). These results are consistent with the recent study that reported a decrease in the serum TNF-α, MCP-1, IL-1β, and IL-6 levels with SAL treatment compared with those without treatment in the rats with DN (Qi et al. 2021).

It has been well demonstrated that the overproduction of reactive oxygen species linked to the altered metabolic pathways in the kidneys is related to the progression of DN (Jha et al. 2016). It also activates a series of redox-sensitive signal pathways leading to the development of DN (Kitada et al. 2011). In the present study, levels of MDA were significantly decreased, whereas the activity of SOD was increased in renal tissues of the rats in the SAL-treated group, suggesting the inhibition of oxidative stress and thus reducing reactive oxygen-free radicals or improving the activity of antioxidants and preventing lipid peroxidation. Oxidative stress in diabetes may also be associated with TNF-α signalling. In chronic inflammatory conditions, TNF-α is involved in insulin resistance and its blockade has shown to improve dyslipidemia and altered glucose tolerance (Popa et al. 2007). The decrease in TNF-α content induced by SAL may be related to the suppression of oxidative stress-induced structural and functional damage to the kidney. On the basis of these results, it can be proposed that SAL has a protective role against DN through the inhibition of oxidative stress.

Molecular mechanisms underlying SAL-mediated protection against DN were examined by studying the expression and phosphorylation patterns of Akt and GSK-3β. SAL induced a significant increase in phosphorylation but not protein expression of Akt and GSK-3β in the rats with DN. The PI3K/Akt pathway is involved in the regulation of a variety of signalling molecules or pathways to inhibit apoptosis, including GSK-3β and Bcl-2/Bax. Recently, SAL showed protective effect against neuronal damage in a mouse model of Alzheimer's disease by activating the PI3K/Akt signalling pathway (Zhang et al. 2016). Activated Akt promotes the phosphorylation of downstream molecules, such as GSK-3, FoxOs, Bad, and P21, thereby mediating glucose metabolism through insulin and growth factor (Sarbassov et al. 2005).

Endoplasmic reticulum stress-induced apoptosis is mediated by PI3K/Akt/GSK-3β signalling in β-cells of the pancreatic islet; thus, the suppression of GSK-3β gene expression prevents apoptosis and further stimulates the replication of rat insulinoma cells (Srinivasan et al. 2005; Mussmann et al. 2007). From the results of the present study, SAL promoted phosphorylation of Akt and GSK-3β, thus inhibiting the biological function of GSK-3β, which is consistent with a previous report suggesting an increase in p-Akt levels in PC12 cells by SAL (Zhang et al. 2012) and p-GSK-3β in mouse hepatocytes (Zheng et al. 2015).

However, this study still has certain limitations. We speculated that SAL might play a protective role in DN through the apoptotic pathway only through TUNEL experiment and the detection of Bax and Bcl-2 protein expression. However, the molecular mechanism of apoptosis is very complex and regulated by a variety of apoptosis-related genes. The Bcl-2 protein family and the caspase family play key roles in apoptosis. The area of focus of this research paper is on protective effect of SAL on DN and not the in-depth study of apoptosis mechanism. Therefore, the effect of SAL on apoptosis and its mechanism needs to be studied in the future.

## Conclusion

The present study supports the use of SAL as a therapeutic agent that has a protective action on renal cells and can promote their survival through the activation of Akt-GSK-3β signalling in the rats with DN. These findings can provide a new therapeutic option not only for type 2 diabetes but also for diabetic complications, such as DN. However, it should be noted that *in vivo* studies may not present an actual depiction of the molecular mechanism occurring in human body.

## Data Availability

The data that support the findings of this study are available from the corresponding authors, DX and MP, upon reasonable request.
